# Waiting Lists for Psychotherapy and Provider Attitudes Toward Low-Intensity Treatments as Potential Interventions: Survey Study

**DOI:** 10.2196/39787

**Published:** 2022-09-16

**Authors:** Allison Peipert, Anne C Krendl, Lorenzo Lorenzo-Luaces

**Affiliations:** 1 Department of Psychological and Brain Sciences Indiana University Bloomington Bloomington, IN United States

**Keywords:** psychotherapy, CBT, cognitive behavioral therapy, behavior therapy, digital mental health, self-help, support group, mental health, digital health, eHealth, low-intensity intervention, survey, waiting list, health system, health care delivery, health care professional, care provider, bibliotherapy, attitude, perception, digital intervention, web-based intervention, depression, anxiety, mental disorder

## Abstract

**Background:**

Common mental disorders, including depression and anxiety, are leading causes of disability worldwide. Digital mental health interventions, such as web-based self-help and other low-intensity treatments (LITs) that are not digital (eg, bibliotherapy), have the potential to reach many individuals by circumventing common barriers present in traditional mental health care. It is unclear how often LITs are used in clinical practice, or whether providers would be interested in their use for treatment waiting lists.

**Objective:**

The aims of this study were to (1) describe current practices for treatment waiting lists, (2) describe providers’ attitudes toward digital and nondigital LITs for patients on a waiting list, and (3) explore providers’ willingness to use digital and nondigital LITs and their decisions to learn about them.

**Methods:**

We surveyed 141 practicing mental health care providers (eg, therapists and psychologists) and provided an opportunity for them to learn about LITs.

**Results:**

Most participants reported keeping a waiting list. Few participants reported currently recommending digital or nondigital LITs, though most were willing to use at least one for patients on their waiting list. Attitudes toward digital and nondigital LITs were neutral to positive. Guided digital and nondigital LITs were generally perceived to be more effective but less accessible, and unguided interventions were perceived to be less effective but more accessible. Most participants selected to access additional information on LITs, with the most popular being web-based self-help.

**Conclusions:**

Results suggest providers are currently not recommending LITs for patients on treatment waiting lists but would be willing to recommend them. Future work should explore barriers and facilitators to implementing digital and nondigital LITs for patients on treatment waiting lists.

## Introduction

Common mental disorders (CMDs), such as depression, anxiety, and insomnia, are leading causes of disability worldwide [1-3] and a significant burden to the health care system [4]. Mental health needs have increased with the COVID-19 pandemic, such that approximately 3 in 10 adults in the United States report clinically elevated anxiety or depression symptoms—a number that may have tripled since 2019 [5]. Effective treatments exist for CMDs, including psychotherapy and pharmacotherapy. However, despite the prevalence of CMDs and the efficacy of existing treatments, few people with mental health concerns receive any form of treatment [6,7].

Barriers related to receiving traditional psychotherapy or other forms of treatment include time availability, lack of financial resources (including insurance coverage), stigma, and low provider availability [6,8]. During the COVID-19 pandemic, many of these barriers have been amplified, particularly provider availability and waiting time for treatment. Increased delays in treatment have been reported across a number of health service areas, including oncology, elective surgery, and general health care [9,10]. Waiting lists are used by providers as the demand for services often surpasses provider capacity. Generally, this involves individuals seeking treatment to be placed on a list prior to receiving a scheduled appointment or scheduling appointments far in the future. Existing work suggests that treatment waiting lists are commonly used for mental health services, but there is substantial variability in their design and management [11]. A relatively agreed-upon feature of waiting lists is that they are associated with a rather long time waiting for psychological services, raising a number of ethical issues, and forcing individuals to seek out alternate services.

Waiting to receive treatment for mental health specifically can have detrimental effects. For example, waiting for treatment can be associated with increased symptom severity, including symptom deterioration [12]. Additionally, a greater waiting time is associated with a lower likelihood of ultimately engaging with the treatment [13]. Finally, the pretreatment waiting times may also be associated to worse patient engagement even once patients initiate treatment [14,15], decreased probability of improvement with treatment, and increased risk of dropout [13,16]. Patients themselves also identify waiting for treatment as a barrier to care, with some studies reporting negative psychological and behavioral outcomes when placed on treatment waiting lists for mental health care [17].

Current efforts to address barriers to mental health treatment have made little impact on the burden of mental health. For example, research in places where the number of mental health providers has increased show public health burdens of mental illness remain unchanged [18]. Furthermore, some researchers report the public health burden of CMDs, such as mood and anxiety disorders, would show little reduction even if the number of providers doubled instantly [6]. These findings suggest that the current model of mental health care needs to change in order to accommodate the burden of CMDs [7].

Various low-intensity treatments (LITs) exist that are relatively scalable and have demonstrated efficacy for depression, anxiety, and other CMDs. Many of these LITs can be used with additional guidance by a professional or paraprofessional (ie, guided) or can be self-guided by the individual user (ie, unguided) and can be accessed through a digital or nondigital platform. Although face-to-face therapy is one of the most well-studied treatments for CMDs, both guided and unguided LITs have been proven to be effective relative to controls like waiting lists and care as usual [19-22]. In general, unguided self-help is better than control groups (eg, sham internet applications and care as usual), and guided self-help is more efficacious than both controls and unguided self-help [23], with guided self-help appearing to have similar efficacy to face-to-face therapy [24]. One commonly studied nondigital LIT is bibliotherapy, a form of self-help using print materials [25]. Meta-analyses of randomized controlled trials of bibliotherapy for depression support its efficacy, yielding large mean effect sizes [19,20]. Guided and unguided digital mental health interventions (DMHIs), including internet-based cognitive behavioral therapy (iCBT) or mental health apps, are effective at treating depression relative to controls like waiting lists and care as usual [24,26]. Together, these studies suggest that LITs provide a tenable and effective treatment alternative for people with CMDs.

Despite research supporting the efficacy of digital and nondigital LITs, it is unclear to what extent they are used in clinical practice [27]. We propose that one place where digital and nondigital LITs may be impactful is in the provision of services to individuals waiting for treatment [28]. The period in which people are waiting for psychological services is an important time for intervention because individuals have already overcome some barriers to treatment seeking, including stigma and the lack of contact with a mental health provider, only to be faced with another barrier (ie, time waiting without receiving services). Schleider et al [28] made a similar observation in a small open trial, where they offered a nondigital LIT to patients on a waiting list. We could not identify a study where provider attitudes toward digital or nondigital LITs for patients on waiting lists were explored.

There is limited research on the use of LITs for patients on waiting lists; however, preliminary studies have supported the feasibility of implementing mental health apps for patients waiting for treatment [29,30]. Other researchers have explored barriers and facilitators of DMHI implementation in clinical practice more broadly. In one study comparing implementers and nonimplementers of iCBT, the two groups differed significantly in their perceived knowledge, confidence in when to recommend iCBT, perception of technical problems, organizational resources, and patient referral process allowing for iCBT inclusion [27]. Thus, there may be differences in the perceptions and attitudes of those who choose to implement versus those who choose not to implement digital and nondigital LITs, such as iCBT, in their practice. However, we know little about general attitudes toward LITs and, specifically, their potential to be implemented in clinical practice for patients on a waiting list. This is important for two reasons. First, extensive work has shown that attitudes predict behavior [31]; thus, negative attitudes toward digital and nondigital LITs may predict reduced willingness to endorse them. Second, identifying the factors that dissociate between willingness and unwillingness to implement digital and nondigital LITs could inform how future work should shape interventions to promote providers’ LIT use, particularly for practices with prohibitively long wait lists.

The aims of our study were the following: (1) describing current practices surrounding waiting lists, including how often they are used and what providers do with individuals on their waiting lists (aim 1); (2) describing providers’ attitudes toward digital and nondigital LITs for patients on their waiting lists (aim 2); and (3) exploring predictors of providers’ willingness to use digital and nondigital LITs and their decisions to learn more about them (aim 3).

## Methods

We surveyed currently practicing mental health care providers on their attitudes toward LITs, including guided and unguided bibliotherapy and DMHIs. Additionally, we included a behavioral task that provided an option for participants to receive additional information about LITs.

### Recruitment and Eligibility

Participants were recruited using a survey link posted via emails to professional organization listserves, specifically the American Psychological Association Division 29—Society for the Advancement of Psychotherapy, the Association for Behavioral and Cognitive Therapies, and social media (ie, Twitter). The survey, which was advertised as a survey on “waiting lists and possible resources,” began with a Study Information Sheet outlining the purpose of the research, eligibility criteria, limits of confidentiality, risks and benefits, and compensation. Participants were eligible to participate if they identified as being (1) over the age of 18 years, (2) a practicing licensed mental health care professional, and (3) currently providing at least 1 hour of clinical services per week. We required participants to have to conduct at least an hour of clinic work to ensure participants were currently providing at least some clinical services, but we did not limit the study to those whose only duties were clinical work.

We received 145 survey responses. Two responses were removed after being determined to be from the same individual. Two responses were removed for not providing responses to all questions. The remaining 141 participants were included in data analysis.

### Ethics Approval

Study procedures were approved by Indiana University Bloomington’s Human Subjects and Institutional Review Board (10503).

### Data

The survey, which can be found in the Open Science Framework website [32], was divided into 4 sections. Section 1 collected demographic information (eg, age, gender, race, and ethnicity) and clinical background (eg, state or country of licensure, level of education, clinical practice setting, psychotherapy theoretical orientation, satisfaction with clinical work, years of clinical experience, and average hours of clinical services provided per week).

Section 2 was designed to address aim 1 of our study. In this section, participants were asked to provide information on their current waiting list practices. This included whether their current clinical setting kept a treatment waiting list (vs not keeping a waiting list or scheduling appointments several months in advance), the estimated time patients spent on a waiting list, the effect of the COVID-19 pandemic on waiting time, and current actions taken for patients on a treatment waiting list.

In section 3, participants were provided with a brief description of the following LITs: unguided bibliotherapy, guided bibliotherapy, unguided web-based self-help, guided web-based self-help, and patient support groups. We chose these LITs because they have been relatively well researched, and research suggests they are effective. Descriptions of each LIT type were provided to ensure that respondents were equally familiar with each intervention. To address aim 2 of our study, for each LIT, respondents were asked to rate their perceived effectiveness (eg, “I believe this option would be effective for patients on a treatment waiting list”), availability (eg, “I believe this option is available and accessible to use with patients on my waiting list”), and willingness to use LIT (eg, “I am willing to use this modality for patients on a waiting list”). Responses were given on a 5-point Likert scale from 1 (strongly disagree) to 5 (strongly agree).

Section 4 addressed aim 3 of our study by asking respondents if they would like to receive any additional information about the following LITs: bibliotherapy, web-based self-help, or patient support groups. They were subsequently offered information on each LIT, and we tracked which participants chose to receive more information (ie, whether they engaged in information-seeking behavior).

### Missing Data

Missingness in demographic and clinical variables was relatively low (0%-1.4%), with the exception of age (13.5%). Missingness in the attitude variables was greater (10.6%-12.1%), reflecting survey dropout (ie, participants who answered no subsequent questions). We completed regression analyses with the original data set and an imputed data set. The latter imputed missing values for all variables included in the regression analyses using a machine learning algorithm with random forests using the R package “missForest” [33].

### Statistical Analysis

Analyses were conducted using the R programming language (version 4.2.1; the R Core Team) [34]. To describe waiting list practices, we present response frequencies and descriptive statistics (aim 1). We compared descriptive statistics and response frequencies of providers’ perceived efficacy, availability and accessibility, as well as willingness to use the different LITs (aim 2).

To explore predictors of willingness to use digital and nondigital LITs (aim 3), we ran 5 linear regressions, one for each LIT under consideration. Willingness to use each specific intervention was regressed on demographic information (ie, gender, age, and education), professional background (ie, theoretical orientation and practice setting), and clinical variables (ie, the use of a waiting list, clinical satisfaction, and clinical hours per week). In these models, an average “willingness” response variable was included in the regression to control for participant willingness to use other interventions, excluding the one being predicted. Regressions were completed using the original data set and the imputed data set.

To explore predictors of information seeking for digital and nondigital LITs (aim 3), we conducted a series of regressions to explore demographic, professional background and attitudinal predictors of requesting additional information. Specifically, we conducted 3 binomial logistic regressions with the dependent variables of requesting additional information (ie, selecting information vs not selecting information) on the following: (1) web-based self-help, (2) bibliotherapy, and (3) patient support groups. Each model included demographic information (eg, gender, education, and age), clinical variables (eg, theoretical orientation, clinical satisfaction, years of experience, clinical hours, and presence of a waiting list), modality-specific attitudes (eg, willingness to use the specific intervention and perceived availability and accessibility of the specific intervention), and controls for overall rating tendency (eg, average willingness to use other interventions and average availability or accessibility of other interventions).

### Transparency and Openness

We report how we determined our sample, all data exclusions, all manipulations, and all measures in the study. All data, analysis code, and research materials are available on the Open Science Framework website [32]. This study’s design and its analysis were not preregistered. No other papers currently use these data.

## Results

### Sample Characteristics

Demographic and clinical variables of the sample are summarized in Table 1. The sample primarily included female, non-Hispanic, White participants. Participants’ average age was about 39 years. Most participants in the sample had a PhD degree and were prescribed to an orientation (eg, cognitive or third-wave behavioral therapy) related to cognitive behavioral therapy (CBT). About half of the participants were employed in a private practice setting with an average of 10 (SD=10.4) years of clinical experience.

**Table 1:** Demographic and clinical variables of 141 providers who responded to a survey of waiting lists and low-intensity treatments.

Demographics		Values
Age (years), mean (SD)		39.2 (10.1)
**Gender, n (%)**		
	Female	92 (65.2)
	Male	45 (31.9)
	Nonbinary	2 (1.4)
	No answer	2 (1.4)
**Race or ethnicity, n (%)**		
	Non-Hispanic White	115 (81.5)
	Non-Hispanic Black	1 (0.7)
	Hispanic	7 (5)
	Asian	6 (4.3)
	AIAN^a^, MENA^b^, NHPI^c^, or other	6 (4.3)
	Multiracial	6 (4.3)
**Education, n (%)**		
	Associate of Arts degree	1 (0.7)
	Bachelor of Arts degree	3 (2.1)
	Master of Arts degree	17 (12.1)
	PhD	99 (70.2)
	PsyD	18 (12.8)
	Other	3 (2.1)
Private practice (vs no private practice), n (%)		71 (50.7)
Clinical orientation—CBT^d^ (vs other), n (%)		125 (88.7)
**Clinical satisfaction, n (%)**		
	Not satisfied	0 (0)
	Slightly satisfied	7 (5)
	Neutral	23 (16.4)
	Very satisfied	80 (57.1)
	Extremely satisfied	30 (21.4)
Clinical experience (years), mean (SD)		10.4 (10.4)
Clinical hours per week, mean (SD)		18.3 (10.9)

^a^AIAN: American Indian or Alaska Native.

^b^MENA: Middle Eastern or North African.

^c^NHPI: Native Hawaiian or Pacific Islander.

^d^CBT: cognitive behavioral therapy.

### Waiting List Characteristics

The majority of survey respondents (n=94, 69.1%) endorsed keeping a formal treatment waiting list (Table 2) with the estimated waiting time averaging about 13 weeks. Others reported not keeping a waiting list but scheduling patients “a couple of months in advance” (n=23, 16.9%). Most of the respondents who endorsed keeping a waiting list also noted their estimated waiting time had been impacted by the COVID-19 pandemic (n=68, 73%) and estimated waiting times around 9 weeks prior to the pandemic.

The majority of participants who kept a waiting list or scheduled patients for months in advance endorsed taking names or contact information, completing an unstructured brief assessment, and providing referrals. Few reported completing a structured assessment or provided brief psychoeducation, and even fewer (n=13-20, <20%) reported providing information on apps, books, or support groups.

**Table 2:** Features of treatment waiting lists for 141 providers who responded to our survey on waiting lists and low-intensity treatments.

Characteristics		Values	Missing values, n (%)
**Waiting list kept, n (%)**			5 (3.5)
	Yes	94 (69.1)	
	No	23 (16.9)	
	No, but scheduling appointments months in advance	19 (14)	
**Impacted by COVID-19, n (%)**			1 (1.1)
	Yes	68 (73.1)	
	No	25 (26.9)	
**Wait time, mean (SD)**			
	Current wait time (weeks)	12.8 (10.6)	
	Pre**–**COVID-19 wait time (weeks)	9.4 (11.7)	
**Waiting list resources used by therapists who kept a waiting list (n=106), n (%)**			7 (6.2)
	Name or contact information	101 (95)	
	Unstructured assessment	77 (72.6)	
	Structured assessment	24 (22.6)	
	Referrals (psychology or psychiatry)	88 (83)	
	Brief psychoeducation	29 (27.4)	
	Books	17 (16)	
	Apps	13 (12.3)	
	Support groups	20 (18.9)	
	Other	9 (8.5)	

### Attitudes Toward Digital and Nondigital Low-Intensity Treatments

**Figure 1:**
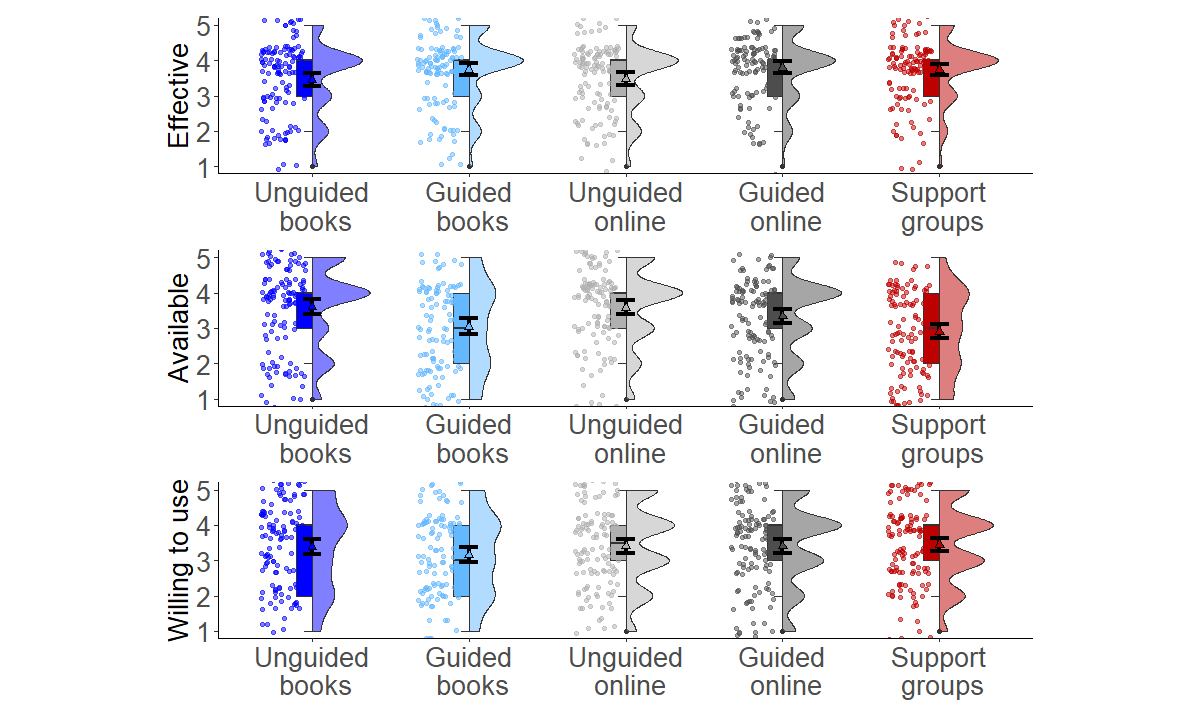
Willingness to use, perceived availability, and perceived efficacy of different low-intensity interventions presented to 141 mental health providers. Box plot indicates median and interquartile range. Triangle and error bars indicate mean values and 95% CI. Violin plot shows distribution of the data.

Figure 1 shows respondents’ ratings of willingness to use, perceived efficacy, and availability of different interventions. Average responses were generally between 3 (“neutral”) and 4 (“somewhat agree”). The exception to this was the perceived low accessibility and availability of patient support groups (mean 2.9, SD 1.2). Average attitudes were highly correlated; specifically, the willingness to use LITs was related to its perceived efficacy (*r*_122_= 0.76, 95% CI 0.67-0.83; *P*<.001).

### Predictors of Willingness to Use an Intervention

We used multiple linear regressions to identify demographic, clinical, waiting list, and attitude variables predicting participants’ ratings of willingness to use a LIT (Table S1 in Multimedia Appendix 1). Across models, averaged willingness to use other LITs predicted the specific likelihood of willingness to use another LIT. For example, the willingness to use unguided bibliotherapy was strongly predicted by the average willingness to use support groups, guided bibliotherapy, and web-based self-help (β=.65, 95% CI 0.45-0.84).

Other predictors of willingness to use a LIT varied across the LITs probed. Compared to individuals in other practice settings, individuals in private practice were more willing to use unguided bibliotherapy (β=.22, 95% CI 0.02-0.42) and less willing to use guided bibliotherapy (β=–.20, 95% CI –0.35 to –0.04). Additionally, individuals who reported a CBT theoretical orientation (vs not reporting one) were more willing to use unguided web-based self-help (β=.22, 95% CI 0.08-0.35). There were no consistent significant predictors of willingness to use support groups. More detailed information is presented in Table S1 in Multimedia Appendix 1.

### Information Seeking About Digital and Nondigital Low-Intensity Treatments

Most (n=85, 70%) respondents indicated they would like to receive additional information about at least one of these modalities: web-based self-help (n=78, 63%), bibliotherapy (n=66, 53%), or support groups (n=61, 50%). We explored demographic, clinical, waiting list, and attitude variables predicting participants’ responses to receive additional information. Overall, there were few strong predictors of information seeking for LITs (Table 3 and Table 4). Willingness to use the specific LIT was predictive of information seeking for web-based self-help (odds ratio [OR]=2.20, 95% CI 1.20-4.28) and bibliotherapy (OR=1.66, 95% CI 1.02-2.80). Relative to individuals in other practice settings, individuals in private practice were more likely to seek information for web-based self-help (OR=5.33, 95% CI 1.48-23.42). There were no consistent predictors of seeking information about support groups.

**Table 3:** Logistic regression analyses predicting providers’ information seeking for low-intensity interventions from demographic, clinical, and practice predictors in original data sets.

Original data (N=104)	Web-based self-help			Bibliotherapy				Support groups		
	OR^a^ (95% CI)	Z^b^	*P* value	OR (95% CI)	Z	*P* value	OR (95% CI)		Z	*P* value
Intercept	0.16 (0.00-8.08)	–0.91	.36	0.56 (0.01-31.65)	–0.28	.78	0.11 (0.00-5.37)		–1.10	.27
Availability (specific)	0.71 (0.41-1.20)	–1.23	.22	0.94 (0.55-1.58)	–0.23	.82	0.98 (0.66-1.43)		–0.12	.90
Willingness (specific)	2.20 (1.20-4.28)	2.45	.01^c^	1.66 (1.02-2.80)	1.99	.05^c^	1.24 (0.77-2.01)		0.87	.38
Availability (general)	1.51 (0.77-3.04)	1.19	.23	1.08 (0.55-2.14)	0.22	.83	0.97 (0.52-1.79)		–0.11	.91
Willingness (general)	1.29 (0.59-2.84)	0.65	.51	1.64 (0.84-3.38)	1.41	.16	1.70 (0.88-3.44)		1.54	.12
Age	0.97 (0.92-1.01)	–1.36	.17	0.99 (0.94-1.04)	–0.46	.64	0.97 (0.93-1.02)		–1.13	.26
Female (vs male)	0.41 (0.12-1.20)	–1.57	.12	0.84 (0.31-2.24)	–0.35	.73	0.69 (0.27-1.75)		–0.78	.44
Doctorate (vs AA/BA/MA^d^)	1.50 (0.30-6.76)	0.52	.60	1.38 (0.33-5.55)	0.45	.65	2.20 (0.56-9.21)		1.12	.26
Keeps a waitlist (vs not)	2.52 (0.64-10.25)	1.33	.18	1.36 (0.41-4.64)	0.50	.62	0.51 (0.14-1.72)		–1.07	.29
Clinical satisfaction (1-5)	0.84 (0.39-1.77)	–0.46	.65	0.56 (0.27-1.10)	–1.64	.10	1.00 (0.51-1.99)		0.01	.99
Clinical hours	1.00 (0.96-1.05)	0.20	.84	1.00 (0.96-1.05)	0.20	.85	0.99 (0.95-1.03)		–0.50	.62
Private practice (vs other)	5.33 (1.48-23.46)	2.40	.01^c^	2.52 (0.84-8.32)	1.60	.11	2.14 (0.72-6.84)		1.34	.18
CBT^e^ (vs other orientation)	0.95 (0.20-4.42)	–0.06	.95	0.41 (0.09-1.74)	–1.18	.24	1.75 (0.41-8.28)		0.74	.46

^a^OR: odds ratio.

^b^*Z* statistic from specific model term in logistic regression.

^c^*P* values are significant when *P*<.05.

^d^AA/BA/MA: Associate of Art degree/ Bachelor of Arts degree/Master of Arts degree.

^e^CBT: cognitive behavioral therapy.

**Table 4:** Logistic regression analyses predicting providers’ information seeking for low-intensity interventions from demographic, clinical, and practice predictors in imputed data sets.

Imputed data (N=141)	Eb-based self-help			Bibliotherapy				Support groups		
	OR^a^ (95% CI)	*Z* ^b^	*P* value	OR (95% CI)	*Z*	*P* value	OR (95% CI)		*Z*	*P* value
Intercept	0.05 (0.00-1.23)	–1.79	.07	0.12 (0.00-3.20)	–1.25	.21	0.17 (0.01-3.42)		–1.15	.25
Availability (specific)	0.79 (0.47-1.27)	–0.96	.34	1.01 (0.62-1.61)	0.03	.98	0.96 (0.67-1.36)		–0.25	.8
Willingness (specific)	1.95 (1.17-3.38)	2.48	.01^c^	1.50 (0.98-2.32)	1.87	.06	1.45 (0.98-2.19)		1.81	.07
Availability (general)	1.74(0.95-3.27)	1.78	.08	1.42 (0.76-2.73)	1.10	.27	1.13 (0.65-1.97)		0.42	.68
Willingness (general)	1.14 (0.59-2.17)	0.40	.69	1.48 (0.81-2.77)	1.27	0.20	1.24 (0.69-2.27)		0.42	.48
Age	0.98 (0.93-1.02)	–1.06	.29	1.00 (0.95-1.04)	–0.15	.88	0.98 (0.94-1.02)		–0.92	.36
Female (vs male)	0.57 (0.21-1.44)	–1.16	.24	1.00 (0.41-2.42)	0.01	.99	0.75 (0.34-1.64)		–0.71	.48
Doctorate (vs AA/BA/MA^d^)	0.79 (0.28-2.14)	–0.46	.65	0.84 (0.33-2.06)	–0.39	.70	0.96 (0.40-2.26)		–0.09	.93
Keeps a waitlist (vs not)	3.32 (1.10-10.30)	2.12	.03^c^	2.63 (0.94-7.73)	1.81	.07	0.91 (0.33-2.47)		–0.18	.86
Clinical satisfaction (1-5)	1.21 (0.66-2.25)	0.62	.54	0.79 (0.44-1.40)	–0.81	.42	1.18 (0.69-2.01)		0.60	.55
Clinical hours	1.00 (0.96-1.05)	0.14	.89	1.01 (0.97-1.05)	0.28	.78	0.99 (0.95-1.03)		–0.55	.58
Private practice (vs other)	2.85 (1.06-8.42)	2.00	.05^c^	1.34 (0.55-3.38)	0.64	.52	1.18 (0.50-2.86)		0.37	.71
CBT^d^ (vs other orientation)	0.75 (0.19-2.59)	–0.45	.65	0.37 (0.09-1.30)	–1.48	.14	1.07 (0.33-3.39)		0.12	.90

^a^OR: odds ratio.

^b^*Z* statistic from specific model term in logistic regression.

^c^*P* values are significant when *P*<.05.

^d^AA/BA/MA: Associate of Art degree/ Bachelor of Arts degree/Master of Arts degree.

^d^CBT: cognitive behavioral therapy.

## Discussion

### Principal Findings

The aims of this study were to describe current waiting list practices (aim 1), describe providers’ attitudes toward digital and nondigital LITs for patients on their waiting lists (aim 2), and explore predictors of providers’ willingness to use digital and nondigital LITs and their decisions to learn more about them (aim 3). Most providers (n=94, 69%) endorsed keeping a treatment waiting list. Among those who said they do not, nearly half (n=19, 45%) reported scheduling patients in the “distant future,” for example, 2-3 months away. Thus, most (n=113, 83%) providers in this sample had an opportunity to use LITs with people waiting for treatment. However, fewer than 20% (n=13-20) reported having recommended books, apps, or support groups for patients on a waiting list. The majority of those who endorsed maintaining a waiting list noted the estimated waiting time for their patients to access treatment was high and had increased since the COVID-19 pandemic. The difference in the average estimated waiting time currently and prior to the pandemic was on average about a month, with a significant increase, suggesting patients have experienced an increased delay in accessing mental health care since the onset of the pandemic.

This study has a number of limitations to consider. First, the generalizability of the study results may be limited due to the relatively small sample size and the representativeness of our study sample, which consisted primarily of CBT-oriented providers. However, our sample was relatively similar to the gender and racial or ethnic demographic characteristics of the psychology workforce in the United States [35], and CBT theoretical orientations have become the most popular among providers [36,37]. Additionally, it is possible that providers who are more interested in digital or nondigital LITs may have been more likely to participate in the study, resulting in a biased rating of attitudes relative to the population of mental health providers. Provider estimates of waiting times may likewise be biased or inaccurate in other ways. Previous research on waiting times for psychological services suggests that average waiting time ranges from 2-3 weeks to 2-3 months [38,39], but this varies across studies. Future studies could explore alternative methods, including the “secret shopper” methods, to get more accurate data on waiting lists and estimated waiting times. Lastly, this study assessed providers’ limited attitudes toward the listed interventions, specifically their perceptions of the intervention efficacy, accessibility, and their willingness to use it. Future research should investigate more detailed perceptions of these interventions through, for example, qualitative interviews to identify specific barriers and facilitators to digital and nondigital LIT implementation and use.

Despite its limitations, this study has notable strengths. To our knowledge, this is the first survey of providers to assess attitudes toward digital and nondigital LITs for patients on a waiting list. Additionally, beyond assessing attitudes, we also provided an opportunity to learn more about digital and nondigital LITs and assessed participants’ decisions to request additional information. Most providers were not currently recommending digital or nondigital LITs for patients on their waiting list, but attitudes toward the interventions were neutral to positive. Most were willing to use at least one intervention for patients on their waiting list. We found no evidence that providers had more positive attitudes regarding digital versus nondigital interventions. Generally, guided interventions were seen as more effective but less accessible than unguided interventions, which, in turn, were seen as less effective but more accessible. Together, these findings support our proposal that dissemination and implementation of digital and nondigital LITs (eg, bibliotherapy or DMHIs) while patients are on waiting lists could be a promising strategy to reduce the burden of untreated CMDs.

Although we found few significant predictors of attitudes and information seeking, we found that practice setting (ie, private practice vs other settings) was a predictor of attitudes and behaviors toward LITs. Individuals in private practice were more willing to use unguided bibliotherapy compared to individuals in other practice settings but were less willing to use guided bibliotherapy and unguided web-based self-help. Interestingly, at the end of our survey, individuals in private practice were more willing to learn about web-based self-help resources compared to individuals in other practice settings. These findings may reflect a knowledge gap wherein individuals in private practice currently do not perceive themselves to know which DMHIs to turn to, a barrier reported by other mental health providers [27,40,41], and hence they are less willing to use them. In addition to private practice setting, CBT theoretical orientation was predictive of willingness to use unguided web-based self-help. This finding may reflect the recent increase in iCBTs in research and practice settings, many of which are self-guided [42,43]. Increasing knowledge about digital and nondigital LITs and disseminating the interventions that individual providers are willing to use may be a useful way of increasing the reach of LITs [44]. One strategy to increase the use of LITs on treatment waiting lists would be to target the dissemination and implementation of digital and nondigital LITs to individuals who already have positive attitudes toward them—in our study, CBT practitioners and people with private practices. Alternatively, researchers could study interventions to increase the willingness to use digital and nondigital LITs by those not predisposed to using them, for example people who are not CBT practitioners.

### Conclusions

We investigated treatment waiting lists and attitudes toward LITs for patients in waiting lists. Most providers appear to keep a waiting list, but most of them do not provide LITs to individuals on their waiting lists. In general, attitudes toward using LITs for patients in waiting lists were positive. Future research should investigate manipulating attitudes toward digital and nondigital LITs as well as structural barriers that may influence their use. For example, regarding individuals in private practice, who may be less likely to recommend guided LITs, qualitative data from our participants and from other studies [45] highlight questions about providers’ legal and ethical liability related to giving LIT guidance for participants on their waiting lists (eg, “I think one might assume a level of risk if participating in guided exercises but not therapy associated with their office”). Additionally, providers may be more, rather than less, likely to recommend LITs to patients with different features (eg, less severe symptoms) [46]. The perceived efficacy of an intervention also seems to be a major correlate of its use, so interventions that educate providers about LIT research are also worth exploring. Future work should clarify the nature of liability when recommending digital and nondigital LITs, as this may be an obstacle to uptake.

## Appendix

Multimedia Appendix 1 Supplementary Table S1.

## Abbreviations

CBT: cognitive behavioral therapy
CMD: common mental disorder
DMHI: digital mental health intervention
iCBT: internet-based cognitive behavioral therapy
LIT: low-intensity treatment
OR: odds ratio

## Data Availability

No other papers currently use these data. Study data, code, and survey materials can be accessed on the Open Science Framework website [32].
