# Supplementary material for: Waiting Lists for Psychotherapy and Provider Attitudes Toward Low-Intensity Treatments as Potential Interventions: Survey Study
Source: JMIR Form Res. 2022 Sep 16;6(9):e39787. doi: 10.2196/39787 (PMC9526124; doi:10.2196/39787)
Supplement: Multimedia Appendix 1 [file formative_v6i9e39787_app1.docx]

| **Table S1.**  Multiple regression analyses of demographic, clinical, and practice predictors of providers’ willingness to use low-intensity interventions in original and imputed datasets | | | | | | | | | | | | | | | | | | | | | | | | | |  | |  |  |  |  |  |  |  |  |  |  |  |  |  |  |  |  |  |  |  |  |  |  |  |
| --- | --- | --- | --- | --- | --- | --- | --- | --- | --- | --- | --- | --- | --- | --- | --- | --- | --- | --- | --- | --- | --- | --- | --- | --- | --- | --- | --- | --- | --- | --- | --- | --- | --- | --- | --- | --- | --- | --- | --- | --- | --- | --- | --- | --- | --- | --- | --- | --- | --- | --- |
|  | Bibliotherapy  Unguided | | | | | Bibliotherapy  Guided | | | | | Online self-help   Unguided | | | | | Online self-help   Guided | | | | | Support groups | | | | | |  |  |  |  |  |  |  |  |  |  |  |  |  |  |  |  |  |  |  |  |  |  |  |  |
|  |  |  |  |  |  |  |  |  |  |  |  |  |  |  |  |  |  |  |  |  |  |  |  |  |  |  |  |  |  |  |  |  |  |  |  |  |  |  |  |  |  |  |  |  |  |  |  |  |  |  |
| Original data (n=104) | B | SE | β | 95% | CI | B | SE | β | 95% | CI | B | SE | β | 95% | CI | B | SE | β | 95% | CI | B | SE | β | 95% | CI | |  |  |  |  |  |  |  |  |  |  |  |  |  |  |  |  |  |  |  |  |  |  |  |  |
| Intercept | -0.10 | 0.86 | -0.08 | -0.36 | 0.20 | -0.03 | 0.70 | -0.17 | -0.39 | 0.06 | 0.08 | 0.66 | 0.06 | -0.17 | 0.29 | 0.66 | 0.65 | 0.12 | -0.11 | 0.35 | 2.97 | 0.73 | 0.08 | -0.20 | 0.36 | |  |  |  |  |  |  |  |  |  |  |  |  |  |  |  |  |  |  |  |  |  |  |  |  |
| Average willingness | **0.83** | **0.12** | **0.65** | **0.45** | **0.84** | **0.88** | **0.10** | **0.65** | **0.50** | **0.80** | **0.81** | **0.09** | **0.64** | **0.49** | **0.79** | **0.78** | **0.09** | **0.63** | **0.48** | **0.77** | **0.63** | **0.11** | **0.55** | **0.37** | **0.74** | |  |  |  |  |  |  |  |  |  |  |  |  |  |  |  |  |  |  |  |  |  |  |  |  |
| Age | 0.01 | 0.01 | 0.07 | -0.10 | 0.25 | 0.00 | 0.01 | -0.02 | -0.16 | 0.12 | -0.01 | 0.01 | -0.07 | -0.22 | 0.07 | 0.00 | 0.01 | -0.02 | -0.17 | 0.12 | -0.01 | 0.01 | -0.08 | -0.25 | 0.10 | |  |  |  |  |  |  |  |  |  |  |  |  |  |  |  |  |  |  |  |  |  |  |  |  |
| Female (vs. male or non-binary) | 0.15 | 0.22 | 0.12 | -0.24 | 0.48 | 0.32 | 0.18 | 0.26 | -0.03 | 0.55 | -0.11 | 0.17 | -0.10 | -0.39 | 0.20 | -0.22 | 0.17 | -0.20 | -0.49 | 0.10 | -0.14 | 0.20 | -0.13 | -0.48 | 0.23 | |  |  |  |  |  |  |  |  |  |  |  |  |  |  |  |  |  |  |  |  |  |  |  |  |
| Doctorate (vs. AA/BA/MA) | 0.08 | 0.32 | 0.02 | -0.15 | 0.20 | -0.16 | 0.26 | -0.05 | -0.19 | 0.10 | -0.06 | 0.24 | -0.02 | -0.16 | 0.12 | -0.18 | 0.24 | -0.05 | -0.20 | 0.09 | 0.00 | 0.28 | 0.00 | -0.17 | 0.17 | |  |  |  |  |  |  |  |  |  |  |  |  |  |  |  |  |  |  |  |  |  |  |  |  |
| Keeps a waitlist | 0.43 | 0.29 | 0.13 | -0.04 | 0.30 | -0.07 | 0.23 | -0.02 | -0.16 | 0.12 | -0.25 | 0.22 | -0.08 | -0.22 | 0.06 | 0.02 | 0.22 | 0.01 | -0.13 | 0.15 | -0.03 | 0.26 | -0.01 | -0.18 | 0.16 | |  |  |  |  |  |  |  |  |  |  |  |  |  |  |  |  |  |  |  |  |  |  |  |  |
| Clinical satisfaction (1-5) | -0.09 | 0.16 | -0.05 | -0.23 | 0.13 | 0.16 | 0.13 | 0.09 | -0.05 | 0.23 | 0.14 | 0.12 | 0.09 | -0.06 | 0.23 | 0.20 | 0.12 | 0.12 | -0.02 | 0.27 | -0.21 | 0.14 | -0.13 | -0.31 | 0.04 | |  |  |  |  |  |  |  |  |  |  |  |  |  |  |  |  |  |  |  |  |  |  |  |  |
| Clinical hours | 0.00 | 0.01 | -0.03 | -0.20 | 0.14 | 0.00 | 0.01 | -0.04 | -0.18 | 0.10 | 0.00 | 0.01 | -0.02 | -0.16 | 0.12 | -0.01 | 0.01 | -0.09 | -0.23 | 0.05 | 0.02 | 0.01 | 0.16 | -0.01 | 0.33 | |  |  |  |  |  |  |  |  |  |  |  |  |  |  |  |  |  |  |  |  |  |  |  |  |
| Private practice (vs. other setting) | **0.55** | **0.25** | **0.22** | **0.02** | **0.42** | **-0.48** | **0.19** | **-0.20** | **-0.35** | **-0.04** | -0.22 | 0.18 | -0.09 | -0.25 | 0.06 | **-0.40** | **0.18** | **-0.17** | **-0.33** | **-0.02** | -0.17 | 0.21 | -0.08 | -0.27 | 0.11 | |  |  |  |  |  |  |  |  |  |  |  |  |  |  |  |  |  |  |  |  |  |  |  |  |
| CBT (vs. other orientation) | 0.22 | 0.34 | 0.05 | -0.11 | 0.22 | -0.07 | 0.27 | -0.02 | -0.15 | 0.12 | **0.81** | **0.26** | **0.22** | **0.08** | **0.35** | 0.08 | 0.26 | 0.02 | -0.12 | 0.16 | -0.57 | 0.30 | -0.16 | -0.33 | 0.01 | |  |  |  |  |  |  |  |  |  |  |  |  |  |  |  |  |  |  |  |  |  |  |  |  |
| Imputed data (n=141) | B | SE | β | 0.95 | CI | B | SE | β | 0.95 | CI | B | SE | β | 0.95 | CI | B | SE | β | 0.95 | CI | B | SE | β | 0.95 | CI | |  |  |  |  |  |  |  |  |  |  |  |  |  |  |  |  |  |  |  |  |  |  |  |  |
| Intercept | 0.18 | 0.69 | 0.00 | -0.14 | 0.14 | 0.58 | 0.54 | 0.05 | -0.12 | 0.12 | 0.36 | 0.56 | 0.00 | -0.12 | 0.12 | 0.72 | 0.55 | 0.00 | -0.12 | 0.12 | 2.47 | 0.59 | 0.00 | -0.14 | 0.14 | |  |  |  |  |  |  |  |  |  |  |  |  |  |  |  |  |  |  |  |  |  |  |  |  |
| Average willingness | **0.77** | **0.11** | **0.57** | **0.41** | **0.73** | **0.85** | **0.09** | **0.62** | **0.49** | **0.75** | **0.78** | **0.09** | **0.59** | **0.45** | **0.73** | **0.75** | **0.09** | **0.57** | **0.44** | **0.71** | **0.60** | **0.10** | **0.51** | **0.35** | **0.67** | |  |  |  |  |  |  |  |  |  |  |  |  |  |  |  |  |  |  |  |  |  |  |  |  |
| Age | 0.01 | 0.01 | 0.05 | -0.12 | 0.21 | 0.00 | 0.01 | -0.03 | -0.17 | 0.10 | -0.01 | 0.01 | -0.06 | -0.20 | 0.08 | 0.00 | 0.01 | -0.02 | -0.16 | 0.12 | -0.01 | 0.01 | -0.11 | -0.27 | 0.05 | |  |  |  |  |  |  |  |  |  |  |  |  |  |  |  |  |  |  |  |  |  |  |  |  |
| Female (vs. male or non-binary) | 0.22 | 0.19 | 0.09 | -0.06 | 0.24 | 0.24 | 0.15 | 0.10 | -0.03 | 0.22 | -0.16 | 0.16 | -0.07 | -0.20 | 0.06 | -0.24 | 0.15 | -0.10 | -0.23 | 0.03 | -0.09 | 0.17 | -0.04 | -0.19 | 0.11 | |  |  |  |  |  |  |  |  |  |  |  |  |  |  |  |  |  |  |  |  |  |  |  |  |
| Doctorate (vs. AA/BA/MA) | 0.09 | 0.21 | 0.03 | -0.13 | 0.19 | -0.23 | 0.17 | -0.09 | -0.23 | 0.04 | 0.26 | 0.17 | 0.11 | -0.03 | 0.25 | -0.13 | 0.17 | -0.06 | -0.19 | 0.08 | 0.05 | 0.19 | 0.02 | -0.14 | 0.18 | |  |  |  |  |  |  |  |  |  |  |  |  |  |  |  |  |  |  |  |  |  |  |  |  |
| Keeps a waitlist | 0.23 | 0.24 | 0.07 | -0.08 | 0.23 | -0.02 | 0.19 | 0.00 | -0.13 | 0.12 | -0.19 | 0.20 | -0.06 | -0.19 | 0.07 | 0.16 | 0.20 | 0.05 | -0.08 | 0.18 | -0.03 | 0.22 | -0.01 | -0.16 | 0.14 | |  |  |  |  |  |  |  |  |  |  |  |  |  |  |  |  |  |  |  |  |  |  |  |  |
| Clinical satisfaction (1-5) | -0.01 | 0.13 | -0.01 | -0.17 | 0.16 | 0.07 | 0.10 | 0.05 | -0.09 | 0.18 | 0.10 | 0.10 | 0.07 | -0.07 | 0.21 | 0.15 | 0.10 | 0.11 | -0.04 | 0.25 | -0.10 | 0.12 | -0.07 | -0.24 | 0.10 | |  |  |  |  |  |  |  |  |  |  |  |  |  |  |  |  |  |  |  |  |  |  |  |  |
| Clinical hours | 0.00 | 0.01 | 0.01 | -0.15 | 0.17 | -0.01 | 0.01 | -0.08 | -0.21 | 0.05 | -0.01 | 0.01 | -0.06 | -0.19 | 0.08 | **-0.02** | **0.01** | **-0.16** | **-0.30** | **-0.02** | **0.02** | **0.01** | **0.17** | **0.01** | **0.32** | |  |  |  |  |  |  |  |  |  |  |  |  |  |  |  |  |  |  |  |  |  |  |  |  |
| Private practice (vs. other setting) | 0.38 | 0.20 | 0.16 | -0.01 | 0.33 | **-0.44** | **0.15** | **-0.19** | **-0.33** | **-0.06** | -0.20 | 0.16 | -0.09 | -0.23 | 0.05 | -0.26 | 0.16 | -0.12 | -0.26 | 0.02 | -0.09 | 0.18 | -0.04 | -0.21 | 0.12 | |  |  |  |  |  |  |  |  |  |  |  |  |  |  |  |  |  |  |  |  |  |  |  |  |
| CBT (vs. other orientation) | 0.11 | 0.28 | 0.03 | -0.12 | 0.18 | -0.04 | 0.22 | -0.01 | -0.13 | 0.11 | **0.62** | **0.23** | **0.18** | **0.05** | **0.30** | 0.11 | 0.22 | 0.03 | -0.10 | 0.16 | -0.46 | 0.25 | -0.14 | -0.29 | 0.01 | |  |  |  |  |  |  |  |  |  |  |  |  |  |  |  |  |  |  |  |  |  |  |  |  |
| Note. B = unstandardized beta, SE = standard error, β = standardized beta, CI = confidence interval, AA = associate’s degree, BA = bachelor’s degree, MA = master’s degree, CBT = cognitive behavioral | | | | | | | | | | | | | | | | | | | | | | | | | | |  |  |  |  |  |  |  |  |  |  |  |  |  |  |  |  |  |  |  |  |  |  |  |  |
